# Patient safety in pre-hospital emergency tracheal intubation: a comprehensive meta-analysis of the intubation success rates of EMS providers

**DOI:** 10.1186/cc11189

**Published:** 2012-02-11

**Authors:** Hans Morten Lossius, Jo Røislien, David J Lockey

**Affiliations:** 1 Department of Research and Development, The Norwegian Air Ambulance Foundation, Holterveien 24, PO Box 94, N-1441 Drøbak, Norway; 2 Department of Surgical Sciences, Faculty of Medicine and Dentistry, University of Bergen, Harald Hårfagres gate 1, PO Box 7804, N-5020 Bergen, Norway; 3 Department of Biostatistics, Institute of Basic Medical Sciences, University of Oslo, 0317 Oslo, Norway; 4 School of Clinical Sciences, University of Bristol & Department of Anaesthesia, North Bristol NHS Trust, Bristol BS16 1LE, UK; 5 London's Air Ambulance, Royal London Hospital, Whitechapel Road, London E1 1BB, UK

## Abstract

**Introduction:**

Pre-hospital airway management is a controversial subject, but there is general agreement that a small number of seriously ill or injured patients require urgent emergency tracheal intubation (ETI) and ventilation. Many European emergency medical services (EMS) systems provide physicians to care for these patients while other systems rely on paramedics (or, rarely, nurses). The ETI success rate is an important measure of provider and EMS system success and a marker of patient safety.

**Methods:**

We conducted a systematic search of Medline and EMBASE to identify all of the published original English-language articles reporting pre-hospital ETI in adult patients. We selected all of the studies that reported ETI success rates and extracted information on the number of attempted and successful ETIs, type of provider, level of ETI training and the availability of drugs on scene. We calculated the overall success rate using meta-analysis and assessed the relationships between the ETI success rate and type of provider and between the ETI success rate and the types of drugs available on the scene.

**Results:**

From 1,070 studies initially retrieved, we identified 58 original studies meeting the selection criteria. Sixty-four per cent of the non-physician-manned services and 54% of the physician-manned services reported ETI success rates but the success rate reporting was incomplete in three studies from non-physician-manned services. Median success rate was 0.905 (0.491, 1.000). In a weighted linear regression analysis, physicians as providers were significantly associated with increased success rates, 0.092 (*P *= 0.0345). In the non-physician group, the use of drug-assisted intubation significantly increased the success rates. All physicians had access to traditional rapid sequence induction (RSI) and, comparing these to non-physicians using muscle paralytics or a traditional RSI, there still was a significant difference in success rate in favour of physicians, 0.991 and 0.955, respectively (*P *= 0.047).

**Conclusions:**

This comprehensive meta-analysis suggests that physicians have significantly fewer pre-hospital ETI failures overall than non-physicians. This finding, which remains true when the non-physicians administer muscle paralytics or RSI, raises significant patient safety issues. In the absence of pre-hospital physicians, conducting basic or advanced airway techniques other than ETI should be strongly considered.

## Introduction

Airway compromise has been identified as a preventable cause of poor outcomes and death in trauma and cardiac arrest patients for many years [1,2]. After arriving in a hospital, the critical and complex intervention of emergency tracheal intubation (ETI) is usually provided by appropriately trained physicians. Most of these physicians are trained anaesthesiologists or emergency physicians trained in anaesthesiology [3,4]. An in-hospital ETI intervention allows administration of drugs that optimize the conditions for tube insertion and minimize physiological derangement and other adverse events [4]. Unsuccessful or poorly conducted ETI can be life threatening and may result in significant complications, such as oesophageal intubation [5], hypoxemia [6], or post-induction cardiac arrest [7].

Rapid sequence induction (RSI) is generally accepted as the technique of choice for securing the airway in seriously ill or injured patients [3,4]. RSI contains three elements: sedation, analgesia and muscle paralysis, all of which are necessary for a safe and successful ETI. The drugs used to perform ETI produce a state of apnoea, can induce hypotension and increase the risk of regurgitation. Using them requires a high level of competence and the ability to deal with any adverse effects. In hospital settings, this requirement usually presupposes the educational level of a specialized physician.

In a pre-hospital setting, the situation is somewhat different. The first Medline- or EMBASE-indexed reports on pre-hospital ETI were published in the mid-to-late1960s [8-13]. Recently, the value of pre-hospital ETI has been seriously questioned [14-17]. Despite many published studies, the benefits of this practice in different patient groups, the skills required by the providers, the effect of different techniques and the alternatives to intubation are less clear now than ever before. The majority of the published papers are based on observational methodologies and are usually considered to be low-quality evidence [18]. Despite the publication of guidelines from Europe and the US that recognize the need for appropriately conducted pre-hospital RSI [19-21] in a small number of patients, the practice is still widely variable between and within countries. In many European countries in which specially trained physicians have participated in pre-hospital EMS services since the late 1950s, RSI is a core component of pre-hospital advanced life support [22-24]. In contrast, some pre-hospital EMS systems in developed countries base their advanced life support entirely on paramedics and/or nurses, and their ETI protocols and procedures depend far less on drug administration [25,26]. A recent systematic review extracted the Utstein airway template variables from studies pertaining to pre-hospital ETI [27]. The majority of the included studies (59.8%) were from North American EMS systems. Of these, 46 (78%) described services in which non-physicians conducted ETI. In contrast, physicians performed the pre-hospital ETIs in 13 (87%) of the 15 non-North American EMS systems. Of the 47 non-physician-manned systems, 25 (53%) performed drug-assisted ETI [27].

As a complex intervention performed by operators with different skill levels in different ways on different patient groups, the effect of pre-hospital ETI on patient outcome is difficult to assess. However, the rate of successful placement of a tracheal tube into the trachea after attempted intubation, particularly after the administration of a muscle paralytic, is recognized as a quality indicator for systems practicing ETI. Although muscle paralytics are administered to facilitate intubation they also render the patient apnoeic and, therefore, make the consequences of failed intubation much more serious.

The aim of this project was to establish whether the published literature indicates a difference in ETI success rates between physician- and non-physician-manned EMS systems. We compared the success rates of non-physicians and physicians and those of non-physicians using different levels of drug assistance. Further, we wanted to explore whether there was a difference in ETI success rates between physicians and the sub-group of non-physicians using a muscle paralytic or a standard RSI.

## Materials and methods

### Identification and selection of studies

We conducted a systematic search of Medline and EMBASE according to the Preferred Reporting Items for Systematic Reviews and Meta-Analyses (PRISMA) guidelines [28]. We identified all original English-language articles published prior to 1 September 2009 that pertained to pre-hospital ETI in adult patients [27]. The studies that investigated paediatric cohorts, that focused on surgical airways and that compared ETI to other airway devices were excluded. The reference lists of the included studies and a recent relevant Cochrane review [17] were inspected to identify any additional relevant studies (see Table 1 for the search strategy).

**Table 1:** Search strategy for identification of relevant studies in Medline and EMBASE

Search terms 'keywords':	
Medline	'Emergency Medical Services' AND 'Intubation, Intratracheal'
Embase	'emergency care' AND 'intubation/or respiratory tract intubation'

**Search terms 'title':**	

Medline	'prehospital' AND 'intubation'
	'pre-hospital' AND 'intubation'
	'out-of-hospital' AND 'intubation'
	'prehospital' AND 'RSI' OR 'Rapid sequence induction'
	'pre-hospital' AND 'RSI' OR 'Rapid sequence induction'
	'out-of-hospital' AND 'RSI' OR 'Rapid sequence induction'
EMBASE	'prehospital' AND 'intubation'
	'pre-hospital' AND 'intubation'
	'out-of-hospital' AND 'intubation'
	'prehospital' AND 'RSI' OR 'Rapid sequence induction'
	'pre-hospital' AND 'RSI' OR 'Rapid sequence induction'
	'out-of-hospital' AND 'RSI' OR 'Rapid sequence induction'

### Study eligibility criteria and data extraction

From the initial search, we selected all of the studies reporting ETI success rates. From these papers, we extracted information on the numbers of attempted ETIs and successful ETIs, type of provider, level of ETI training and drug availability on scene. The providers were categorized into two groups: physician and non-physician. The use of drugs was categorized into three groups: 1) no drugs available; 2) analgesics, anaesthetics, or a combination; and 3) muscle paralytics, with or without co-administration of analgesics and anaesthetics, or a standard RSI.

### Statistical meta-analyses

The ETI success rates are reported as medians (range) unless stated otherwise. The individual and overall success rate is presented in a forest plot and the overall success rate was calculated using a random effects meta-analysis for proportions. The analysis did not consider the number of ETI attempts before success was achieved.

To assess the relationships between the ETI success rate and provider type, and between the ETI success rate and types of drugs available on the scene, we performed a weighted univariate linear regression analysis with the ETI success rate as the dependent variable and drug availability and provider type as categorical independent variables. The regression was weighted by the size of each study, that is, by the number of intubation attempts. As all physicians use RSI (drug group '3'), a multiple regression model would have been degenerate and was not performed. All the tests were two-tailed, and statistical significance was indicated by *P *< 0.05.

The data were analysed using R 2.12 (The R Foundation for Statistical Computing, Vienna, Austria) [29].

### Study ethics

As a meta-analysis based on a systematic literature review, this study did not require approval from The Regional Committee for Research Ethics or the National Social Science Services.

## Results

**Figure 1:**
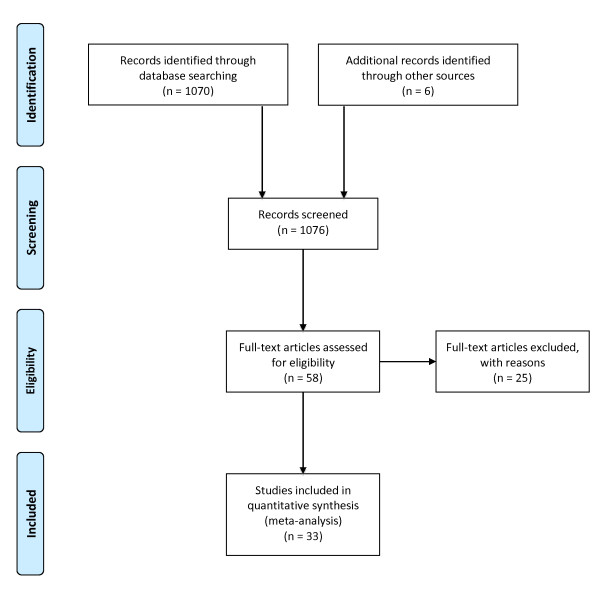
**Search diagram according to the PRISMA statement**.

From 1,070 studies initially retrieved through the systematic search, we identified 58 original studies that met the inclusion criteria. Of these, 45 (78%) were studies of non-physician-manned services (paramedic- or paramedic/nurse-manned). Twenty-nine (64%) of the 45 non-physician-manned services and seven (54%) of the 13 physician-manned services reported ETI success rates. The success rate reporting was incomplete in three studies from non-physician-manned services, leaving 33 studies for the final analysis (Figure 1). An overview of the included studies is shown in Additional file 1.

**Figure 2:**
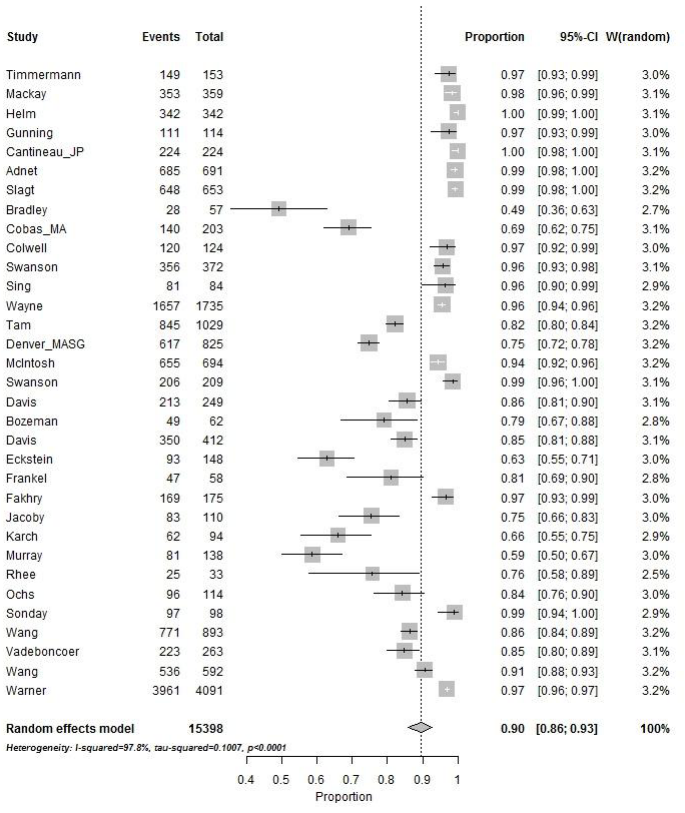
**Forest plot of 33 studies reporting success rate after pre-hospital emergency tracheal intubation**. Individual study estimates of success rates and corresponding 95% CIs.

In total, ETI was attempted in 15,398 patients: 2,536 by physicians and 12,862 by non-physicians. The median (range) reported success rate was 0.905 (0.491, 1.000) (Figure 2). The estimated overall (95% CI) ETI success rate was 0.927 (0.882, 0.961). Figure 2 presents the individual study estimates and corresponding 95% CIs.

When comparing physicians to non-physicians, the corresponding median (range) ETI success rates were 0.991 (0.973, 1.000) versus 0.849 (0.491, 0.990).

All seven physician-manned services reporting success rates also reported drugs available on the scene (all used standard RSI). Of the 26 non-physician-manned services reporting success rates, 19 (73%) reported drugs available on scene, leaving seven services reporting no use of drugs (drug group 1). Of the 19 services reporting use of drugs, six had analgesics, anaesthetics or a combination available (drug group 2), and 13 reported having muscle paralytics, with or without analgesics or anaesthetics, or standard RSI available (drug group 3). In drug groups 1, 2 and 3, the reported median (range) ETI success rates for non-physicians were 0.675 (0.491, 0.968), 0.810 (0.755, 0.905) and 0.967 (0.758, 1.000), respectively.

In weighted linear regression analysis, having physician providers was significantly associated with an increased success rate: 0.092 (0.007, 0.176), *P *= 0.0345. Similarly, drug groups 2 and 3 were significantly associated with an increased success rate: 0.108 (0.033, 0.183), *P *= 0.006 and 0.199 (0.147, 0.252), *P *< 0.001, respectively.

When comparing physician to drug group 3 (muscle paralytics, with or without analgesics or anaesthetics, or standard RSI available) non-physician success rates, there still was a significant difference in favour of physicians: 0.991 (0.974,1.000) and 0.955 (0.758,0.990), respectively (*P *= 0.047).

## Discussion

Airway management in pre-hospital care is complex. Although ETI is only required in a small number of critically ill or injured patients [30,31], it is a well-established tool in pre-hospital EMS services. The procedure carries a risk of severe adverse events if not performed correctly [32] and its providers must be both technically competent to perform the procedure and capable of making decisions and initiating treatments to prevent or treat complications. The ETI success rate is only one component of successful pre-hospital airway management, but a system that performs ETI should strive for a high success rate.

This meta-analysis demonstrates that when non-physicians attempt pre-hospital ETI, they have significantly higher intubation failure rates than do physicians. The ETI failure rate of physician-manned services was on average one out of 100 patients, whereas services manned by non-physicians failed on average in 15 out of 100 patients.

This overall comparison between physician- and non-physician-manned services is important. There is undoubtedly considerable variation in the experience and skill levels covered by both the term 'physicians' and the term 'non-physicians'. The exposure to situations requiring ETI in the pre-hospital setting is, in most EMS services, relatively rare and it can be argued that it is often insufficient to maintain necessary skills. Nevertheless, physicians operating in pre-hospital EMS are likely to have had more training and have performed a greater number of intubations than non-physicians due to their in-hospital clinical activity. In the in-hospital setting, emergency physicians and anaesthesiologists often perform emergency and elective intubations on a regular basis.

Much more relevant to patient safety is the comparison between physicians and non-physicians when muscle paralytics have been administered to facilitate intubation. Failure to achieve intubation after rendering a patient apnoeic has major safety implications and carries a risk of hypoxic brain injury and death [33]. Reports of ETI failure rates of over 15% after administering muscle paralytics are not uncommon in non-physician systems [34-36]. This high failure rate has been previously highlighted [37]; it is not only unthinkable in hospital practice, but is unacceptable in any area of practice. Even though the inclusion of muscle paralytics in non-physician-manned EMS services providing ETI appears to significantly improve intubation success rates it also results in five patients in every100 being rendered apnoeic with an unsecured airway after failed intubation.

The precise clinical implications of our findings are difficult to assess. Failed intubations in hospitals have been subject to considerable analysis, which may give an indication of the consequences of failed pre-hospital intubation. A recent study [38] collected reports of major airway management complications during anaesthesia (death, brain injury, emergency surgical airway, and unanticipated intensive care unit admission) from all of the UK National Health Service hospitals over one year. Difficult or delayed intubation, failed intubation, and 'can't intubate, can't ventilate' accounted for 39% of all such events. In a US study of 179 closed claims arising from managing difficult airways, the majority (67%) of the incidents resulting in death or brain damage involved the induction phase of anaesthesia and were clearly associated with intubation difficulties [33]. It seems that failed intubation is closely associated with the most devastating complications of airway management. In healthcare risk assessment, the significance of a failed intubation and its consequences can be assessed by answering a few key questions [39]: what can go wrong; how bad is it; how likely is it to occur; and what can we do about it? We know what can go wrong in failed pre-hospital intubation and we know the consequences that fall into the potentially 'catastrophic' category (death or severe disability). When constructing a 'risk matrix', the only other key information required is the frequency or likelihood of the event occurring in a given system. The results of combining the potentially severe consequences from failed intubation assisted by muscle paralytics and our observed frequency of failed intubation in 'non-physician' systems falls into an 'extreme risk' category in the NHS National Patient Safety Agency's patient safety guidance risk matrix [39]. 'Extreme risk' requires urgent action by the highest level of an organization [39]. If the data presented in this review were used in the planning phase of a study comparing the outcomes of pre-hospital airway management in physician and non-physician systems, ethical approval would be difficult to obtain.

One of the principles of recent pre-hospital anaesthesia guidelines is that patients undergoing pre-hospital anaesthesia should have the same standards of care and safety that they would receive in an emergency department [20]. This review suggests that physician-conducted pre-hospital intubation is associated with high levels of success that are similar to those reported in US emergency departments [40]. ETI attempted without drugs has not been associated with improved outcomes in cardiac arrest patients [41] and is only likely to be achieved in trauma patients with a high probability of mortality [42].

In previous studies of failed intubations in paramedic systems [43], it has been suggested that insufficient training of the operators rather than their professional status may be responsible for the poor outcomes. Efforts have been made to train non-physicians in critical care [44,45]. Equipping non-physicians with drugs and training them to conduct RSI raises a number of difficulties that need to be overcome. Both training and skill retention are likely to be difficult, as using elective anaesthetic techniques without the need for intubation is becoming more frequent [45,46]. It is also important to ensure that the individuals trained to perform the complex RSI procedure are matched with the few patients that require it and to provide the considerable resources necessary to run such programs. If all of these obstacles are overcome and significant resources are provided to train non-physicians to a high level, what failure rates can be expected? We compared the success rates of non-physicians trained to perform RSI with the success rates of physician-manned services and found that there was still a significant difference. The 2009 study by Warner *et al*. [14] described an exceptionally high level of training, supervision and re-certification and reported on a relatively high number of ETIs. The paramedics were trained in a university training program with 2,500 hours of classroom, laboratory and field experience. Their ETI skills were developed through lectures, intensive mannequin training and experience with patients in the operating room. Field ETIs were then attempted with strict direct supervision and medical oversight. The paramedics also participated in a comprehensive recertification program every two years. A minimum of twelve uncomplicated tracheal intubations per year was required for recertification, and failure to achieve this standard resulted in returning to the operating room for further supervised training. Despite their high level of training, the paramedics in this program still failed to intubate 3 out of 100 patients after administering muscle paralytics (three times the failure rate of physicians), which raises significant patient safety issues.

## Limitations

The results of the analysis in this study must be interpreted with caution due to the small numbers in the patient sub-groups, although the findings remain significant and have narrow CIs. Selection bias, missing cases, and reporting bias in publishing may be conducive to including studies not necessarily representative of real-time clinical activity and performance. The long time frame for including studies and the variance in the reported success rate may diminish this.

This review did not consider a number of other factors that may contribute to poor outcome. Sub-optimally performed ETI, such as multiple intubation attempts, hyper- or hypoventilation and unrecognized oesophageal intubation, may be critical to outcomes and not reflected by intubation success rates. A high rate of undetected oesophageal intubation has been reported in non-physician systems and continues even after introducing easily used carbon dioxide detection equipment [5]. We also did not examine whether non-physicians are better or worse than physicians at managing the consequences of failed ETI. Even in physician-manned services, lack of training and sub-optimal recognition of the indications for advanced airway management may influence outcomes [47-49]. A recent report from a European group of pre-hospital critical care researchers and physicians identified pre-hospital airway management as a prioritized area for future research [50].

## Conclusions

The results of this review suggest that EMS systems in which non-physicians perform ETIs have significantly more failed intubations than systems in which physicians perform ETIs. This increase persists in non-physician systems where muscle paralytics are used and where comprehensive training is provided. If pre-hospital anaesthesia is to be conducted, it should be performed to the highest standards, which includes an intubation success rate close to 100%. This review suggests that this level of performance is currently found only in physician-manned services. Substituting existing physicians with even well-trained non-physicians brings with it significant patient safety issues. It may be that where pre-hospital EMS-physicians are not available, concentrating on basic and advanced airway management techniques other than ETI should be strongly considered in a highly performing EMS system.

## Key messages

• Pre-hospital emergency tracheal intubation (ETI) is provided by both physicians and non-physicians, and the published studies report a wide range in success rates from different emergency medical service (EMS) systems.

• EMS-physicians have ETI success rates close to 100% and significantly higher than non-physicians.

• ETI is a potentially hazardous intervention, especially when conducted with muscle paralytics, and failed intubation increases the risk of severe adverse events and fatal outcome.

• In the absence of pre-hospital physicians, conducting basic and advanced airway techniques other than ETI should be strongly considered.

## Abbreviations

ETI: emergency tracheal intubation; EMS: emergency medical services; RSI: rapid sequence induction

## Competing interests

The authors declare that they have no competing interests.

## Authors' contributions

HML and DJL conceived and planned the study. HML performed the literature review and JR performed the meta-analysis. All authors contributed to writing a draft, and HML wrote the final manuscript, which was read and approved by all the authors.

## Supplementary Material

Additional file 1 **Overview of included studies**. Aim of study, EMS manning, drugs available on scene, number of ETI attempts, number of ETI's successfully conducted, and reported success rate extracted from the 33 reviewed studies. EMS, emergency medical service; ETI, emergency tracheal intubation; RSI, rapid sequence induction. Click here for file

## References

[B1] Anderson ID Woodford M de Dombal FT Irving M Retrospective study of 1000 deaths from injury in England and Wales BMJ 1988 296 1305 1308 10.1136/bmj.296.6632.1305 3133060 PMC2545772

[B2] Esposito TJ Sanddal ND Hansen JD Reynolds S Analysis of preventable trauma deaths and inappropriate trauma care in a rural state J Trauma 1995 39 955 962 10.1097/00005373-199511000-00022 7474014

[B3] Graham CA Advanced airway management in the emergency department: what are the training and skills maintenance needs for UK emergency physicians? Emerg Med J 2004 21 14 19 10.1136/emj.2003.003368 14734367 PMC1756338

[B4] Jensen AG Callesen T Hagemo JS Hreinsson K Lund V Nordmark J Scandinavian clinical practice guidelines on general anaesthesia for emergency situations Acta Anaesthesiol Scand 2010 54 922 950 10.1111/j.1399-6576.2010.02277.x 20701596

[B5] Wirtz DD Ortiz C Newman DH Zhitomirsky I Unrecognized misplacement of endotracheal tubes by ground prehospital providers Prehosp Emerg Care 2007 11 213 218 10.1080/10903120701205935 17454811

[B6] Davis DP Dunford JV Poste JC Ochs M Holbrook T Fortlage D Size MJ Kennedy F Hoyt DB The impact of hypoxia and hyperventilation on outcome after paramedic rapid sequence intubation of severely head-injured patients J Trauma 2004 57 1 8; discussion 8-10 10.1097/01.TA.0000135503.71684.C8 15284540

[B7] Bernard SA Nguyen V Cameron P Masci K Fitzgerald M Cooper DJ Walker T Std BP Myles P Murray L Taylor D Smith K Patrick I Edington J Bacon A Rosenfeld JV Judson R Prehospital rapid sequence intubation improves functional outcome for patients with severe traumatic brain injury: a randomized controlled trial Ann Surg 2010 252 959 965 10.1097/SLA.0b013e3181efc15f 21107105

[B8] Fischer G [Intubation or tracheotomy at the place of accident (at the same time a contribution to the equipment of the physician's emergency kit)] Z Arztl Fortbild 1965 59 1314 1316 5888485

[B9] Lick RF [Medical first aid in accidents] Munch Med Wochenschr 1969 111 340 345 5819135

[B10] Lick RF Schlafer H Martens HL Balser D [Medical and technical first aid at the scene of accident] Munch Med Wochenschr 1969 111 361 369 5819138

[B11] Lick RF Schlafer H Martens HL Seegerer K Holle F [Two and a half years of emergency medical service in Munich] Munch Med Wochenschr 1969 111 356 361 5819137

[B12] Bruckner W Lick R [First aid treatment of the injured at the accident site] Med Klin 1969 64 1019 1021 4895298

[B13] Baskett PJ Zorab JS Priorities in the immediate care of roadside and other traumatic casualties Anaesthesia 1975 30 80 87 10.1111/j.1365-2044.1975.tb00802.x 1115344

[B14] Spaite DW Criss EA Out-of-hospital rapid sequence intubation: are we helping or hurting our patients? Ann Emerg Med 2003 42 729 730 10.1016/S0196-0644(03)00822-9 14634594

[B15] Bernard SA Paramedic intubation of patients with severe head injury: a review of current Australian practice and recommendations for change Emerg Med Australas 2006 18 221 228 10.1111/j.1742-6723.2006.00850.x 16712531

[B16] Stiell IG Nesbitt LP Pickett W Munkley D Spaite DW Banek J Field B Luinstra-Toohey L Maloney J Dreyer J Lyver M Campeau T Wells GA OPALS Study Group The OPALS Major Trauma Study: impact of advanced life-support on survival and morbidity.[see comment] CMAJ 2008 178 1141 1152 10.1503/cmaj.071154 18427089 PMC2292763

[B17] Lecky F Bryden D Little R Tong N Moulton C Emergency intubation for acutely ill and injured patients Cochrane Database Syst Rev 2008 16 CD001429 18425873 10.1002/14651858.CD001429.pub2 PMC7045728

[B18] Guyatt GH Oxman AD Vist GE Kunz R Falck-Ytter Y Alonso-Coello P Schunemann HJ GRADE: an emerging consensus on rating quality of evidence and strength of recommendations BMJ 2008 336 924 926 10.1136/bmj.39489.470347.AD 18436948 PMC2335261

[B19] Wang HE O'Connor RE Domeier RM Prehospital rapid-sequence intubation Prehosp Emerg Care 2001 5 40 48 10.1080/10903120190940317 11194068

[B20] Pre-hospital Anaesthesia: A safety guideline http://www.aagbi.org/sites/default/files/prehospital_glossy09.pdf

[B21] Berlac P Hyldmo PK Kongstad P Kurola J Nakstad AR Sandberg M Pre-hospital airway management: guidelines from a task force from the Scandinavian Society for Anaesthesiology and Intensive Care Medicine Acta Anaesthesiol Scand 2008 52 897 907 10.1111/j.1399-6576.2008.01673.x 18702752

[B22] Adnet F Lapostolle F International EMS systems: France Resuscitation 2004 63 7 9 10.1016/j.resuscitation.2004.04.001 15451580

[B23] Roessler M Zuzan O EMS systems in Germany Resuscitation 2006 68 45 49 10.1016/j.resuscitation.2005.08.004 16401522

[B24] Langhelle A Lossius HM Silfvast T Bjornsson HM Lippert FK Ersson A Soreide E International EMS Systems: the Nordic countries Resuscitation 2004 61 9 21 10.1016/j.resuscitation.2003.12.008 15081176

[B25] Hubble MW Brown L Wilfong DA Hertelendy A Benner RW Richards ME A meta-analysis of prehospital airway control techniques part I: orotracheal and nasotracheal intubation success rates Prehosp Emerg Care 2010 14 377 401 10.3109/10903121003790173 20507222

[B26] Black JJ Davies GD International EMS systems: United Kingdom Resuscitation 2005 64 21 29 10.1016/j.resuscitation.2004.10.004 15629551

[B27] Lossius HM Sollid SJ Rehn M Lockey DJ Revisiting the value of pre-hospital tracheal intubation: an all time systematic literature review extracting the Utstein airway core variables Crit Care 2011 15 R26 10.1186/cc9973 21244667 PMC3222062

[B28] Moher D Liberati A Tetzlaff J Altman DG Preferred reporting items for systematic reviews and meta-analyses: the PRISMA statement BMJ 2009 339 b2535 10.1136/bmj.b2535 19622551 PMC2714657

[B29] R Development Core Team R: A language and environment for statistical computing 2008 Vienna, Austria: R Foundation for Statistical Computing

[B30] Sise MJ Shackford SR Sise CB Sack DI Paci GM Yale RS O'Reilly EB Norton VC Huebner BR Peck KA Early intubation in the management of trauma patients: indications and outcomes in 1,000 consecutive patients J Trauma 2009 66 32 39; discussion 39-40 10.1097/TA.0b013e318191bb0c 19131803

[B31] Trauma: Who cares? http://www.ncepod.org.uk/2007report2/Downloads/SIP_summary.pdf#search='trauma'

[B32] Lin CC Chen KF Shih CP Seak CJ Hsu KH The prognostic factors of hypotension after rapid sequence intubation Am J Emerg Med 2008 26 845 851 10.1016/j.ajem.2007.11.027 18926339

[B33] Peterson GN Domino KB Caplan RA Posner KL Lee LA Cheney FW Management of the difficult airway: a closed claims analysis Anesthesiology 2005 103 33 39 10.1097/00000542-200507000-00009 15983454

[B34] Cobas MA De la Pena MA Manning R Candiotti K Varon AJ Prehospital intubations and mortality: a level 1 trauma center perspective.[see comment] Anesth Analg 2009 109 489 493 10.1213/ane.0b013e3181aa3063 19608824

[B35] Blostein PA Koestner AJ Hoak S Failed rapid sequence intubation in trauma patients: esophageal tracheal combitube is a useful adjunct J Trauma 1998 44 534 537 10.1097/00005373-199803000-00021 9529185

[B36] Davis DP Valentine C Ochs M Vilke GM Hoyt DB The Combitube as a salvage airway device for paramedic rapid sequence intubation Ann Emerg Med 2003 42 697 704 10.1016/S0196-0644(03)00396-2 14581924

[B37] Herff H Wenzel V Lockey D Prehospital intubation: the right tools in the right hands at the right time Anesth Analg 2009 109 303 305 10.1213/ane.0b013e3181ad8a1e 19608796

[B38] Cook TM Woodall N Frerk C Major complications of airway management in the UK: results of the Fourth National Audit Project of the Royal College of Anaesthetists and the Difficult Airway Society. Part 1: anaesthesia Br J Anaesth 2011 106 617 631 10.1093/bja/aer058 21447488

[B39] Agency TNPS Healthcare Risk Assessment made Easy 2007 London: The National Patient Safety Agency

[B40] Dunham CM Barraco RD Clark DE Daley BJ Davis FE Gibbs MA Knuth T Letarte PB Luchette FA Omert L Weireter LJ Wiles CE EAST Practice Management Guidelines Work Group Guidelines for emergency tracheal intubation immediately after traumatic injury.[see comment] J Trauma 2003 55 162 179 10.1097/01.ta.0000083335.93868.2c 12855901

[B41] Deakin CD Nolan JP Soar J Sunde K Koster RW Smith GB Perkins GD European Resuscitation Council Guidelines for Resuscitation 2010 Section 4. Adult advanced life support Resuscitation 2010 81 1305 1352 10.1016/j.resuscitation.2010.08.017 20956049

[B42] Lockey D Davies G Coats T Survival of trauma patients who have prehospital tracheal intubation without anaesthesia or muscle relaxants: observational study BMJ 2001 323 141 11463683 10.1136/bmj.323.7305.141 PMC34726

[B43] Wang HE Sweeney TA O'Connor RE Rubinstein H Failed prehospital intubations: an analysis of emergency department courses and outcomes Prehosp Emerg Care 2001 5 134 141 10.1080/10903120190939995 11339722

[B44] Critical care paramedics: delivering enhanced pre-hospital trauma and resuscitation care: a cost-effective approach http://www.nhsconfed.org/Publications/reports/Pages/Critical-care-paramedics.aspx

[B45] Warner KJ Carlbom D Cooke CR Bulger EM Copass MK Sharar SR Paramedic training for proficient prehospital endotracheal intubation Prehosp Emerg Care 2010 14 103 108 10.3109/10903120903144858 19947874

[B46] Deakin CD King P Thompson F Prehospital advanced airway management by ambulance technicians and paramedics: is clinical practice sufficient to maintain skills? Emerg Med J 2009 26 888 891 10.1136/emj.2008.064642 19934141

[B47] Sollid SJ Heltne JK Soreide E Lossius HM Pre-hospital advanced airway management by anaesthesiologists: is there still room for improvement? Scand J Trauma Resusc Emerg Med 2008 16 2 10.1186/1757-7241-16-2 18957064 PMC2556637

[B48] Sollid SJ Lossius HM Nakstad AR Aven T Soreide E Risk assessment of pre-hospital trauma airway management by anaesthesiologists using the predictive Bayesian approach Scand J Trauma Resusc Emerg Med 2010 18 22 10.1186/1757-7241-18-22 20409306 PMC2873366

[B49] Sollid SJ Lossius HM Soreide E Pre-hospital intubation by anaesthesiologists in patients with severe trauma: an audit of a Norwegian helicopter emergency medical service Scand J Trauma Resusc Emerg Med 2010 18 30 10.1186/1757-7241-18-30 20546578 PMC2903496

[B50] Fevang E Lockey D Thompson J Lossius HM The top five research priorities in physician-provided pre-hospital critical care: a consensus report from a European research collaboration Scand J Trauma Resusc Emerg Med 2011 19 57 10.1186/1757-7241-19-57 21996444 PMC3204240

[B51] Adnet F Jouriles NJ Le Toumelin P Hennequin B Taillandier C Rayeh F Couvreur J Nougiere B Nadiras P Ladka A Fleury M Survey of out-of-hospital emergency intubations in the French prehospital medical system: a multicenter study Ann Emerg Med 1998 32 454 460 10.1016/S0196-0644(98)70175-1 9774930

[B52] Bozeman WP Kleiner DM Huggett V A comparison of rapid-sequence intubation and etomidate-only intubation in the prehospital air medical setting Prehosp Emerg Care 2006 10 8 13 10.1080/10903120500366854 16418085

[B53] Bradley JS Billows GL Olinger ML Boha SP Cordell WH Nelson DR Prehospital oral endotracheal intubation by rural basic emergency medical technicians Ann Emerg Med 1998 32 26 32 10.1016/S0196-0644(98)70095-2 9656945

[B54] Cantineau JP Tazarourte K Merckx P Martin L Reynaud P Berson C Bertrand C Aussavy F Lepresle E Pentier C Duvaldestin P [Tracheal intubation in prehospital resuscitation: importance of rapid-sequence induction anesthesia] Ann Fr Anesth Reanim 1997 16 878 884 10.1016/S0750-7658(97)89837-1 9750618

[B55] Colwell CB McVaney KE Haukoos JS Wiebe DP Gravitz CS Dunn WW Bryan T An evaluation of out-of-hospital advanced airway management in an urban setting Acad Emerg Med 2005 12 417 422 10.1111/j.1553-2712.2005.tb01542.x 15860695

[B56] Davis DP Ochs M Hoyt DB Bailey D Marshall LK Rosen P Paramedic-administered neuromuscular blockade improves prehospital intubation success in severely head-injured patients J Trauma 2003 55 713 719 10.1097/01.TA.0000037428.65987.12 14566128

[B57] Davis DP Vadeboncoeur TF Ochs M Poste JC Vilke GM Hoyt DB The association between field Glasgow Coma Scale score and outcome in patients undergoing paramedic rapid sequence intubation J Emerg Med 2005 29 391 397 10.1016/j.jemermed.2005.04.012 16243194

[B58] Denver MASG A prospective multicenter evaluation of prehospital airway management performance in a large metropolitan region Prehosp Emerg Care 2009 13 304 310 10.1080/10903120902935280 19499465

[B59] Eckstein M Chan L Schneir A Palmer R Effect of prehospital advanced life support on outcomes of major trauma patients J Trauma 2000 48 643 648 10.1097/00005373-200004000-00010 10780596

[B60] Fakhry SM Scanlon JM Robinson L Askari R Watenpaugh RL Fata P Hauda WE Trask A Prehospital rapid sequence intubation for head trauma: conditions for a successful program J Trauma 2006 60 997 1001 10.1097/01.ta.0000217285.94057.5e 16688061

[B61] Frankel H Rozycki G Champion H Harviel JD Bass R The use of TRISS methodology to validate prehospital intubation by urban EMS providers Am J Emerg Med 1997 15 630 632 10.1016/S0735-6757(97)90174-1 9375541

[B62] Gunning M O'Loughlin E Fletcher M Crilly J Hooper M Ellis DY Emergency intubation: a prospective multicentre descriptive audit in an Australian helicopter emergency medical service Emerg Med J 2009 26 65 69 10.1136/emj.2008.059345 19104110

[B63] Helm M Hossfeld B Schafer S Hoitz J Lampl L Factors influencing emergency intubation in the pre-hospital setting--a multicentre study in the German Helicopter Emergency Medical Service Br J Anaesth 2006 96 67 71 16311285 10.1093/bja/aei275

[B64] Jacoby J Heller M Nicholas J Patel N Cesta M Smith G Jacob S Reed J Etomidate versus midazolam for out-of-hospital intubation: a prospective, randomized trial Ann Emerg Med 2006 47 525 530 10.1016/j.annemergmed.2005.12.009 16713778

[B65] Karch SB Lewis T Young S Hales D Ho CH Field intubation of trauma patients: complications, indications, and outcomes Am J Emerg Med 1996 14 617 619 10.1016/S0735-6757(96)90073-X 8906755

[B66] Mackay CA Terris J Coats TJ Prehospital rapid sequence induction by emergency physicians: is it safe? Emerg Med J 2001 18 20 24 10.1136/emj.18.1.20 11310456 PMC1725520

[B67] McIntosh SE Swanson ER McKeone A Barton ED Location of airway management in air medical transport Prehosp Emerg Care 2008 12 438 442 10.1080/10903120802301518 18924006

[B68] Murray JA Demetriades D Berne TV Stratton SJ Cryer HG Bongard F Fleming A Gaspard D Prehospital intubation in patients with severe head injury J Trauma 2000 49 1065 1070 10.1097/00005373-200012000-00015 11130490

[B69] Ochs M Davis D Hoyt D Bailey D Marshall L Rosen P Paramedic-performed rapid sequence intubation of patients with severe head injuries.[see comment] Ann Emerg Med 2002 40 159 167 10.1067/mem.2002.126397 12140494

[B70] Rhee KJ O'Malley RJ Neuromuscular blockade-assisted oral intubation versus nasotracheal intubation in the prehospital care of injured patients Ann Emerg Med 1994 23 37 42 10.1016/S0196-0644(94)70005-2 8273956

[B71] Sing RF Rotondo MF Zonies DH Schwab CW Kauder DR Ross SE Brathwaite CC Rapid sequence induction for intubation by an aeromedical transport team: a critical analysis Am J Emerg Med 1998 16 598 602 10.1016/S0735-6757(98)90227-3 9786546

[B72] Slagt C Zondervan A Patka P de Lange JJ A retrospective analysis of the intubations performed during 5 years of helicopter emergency medical service in Amsterdam Air Med J 2004 23 36 37 15337954 10.1016/j.amj.2004.06.004

[B73] Sonday CJ Axelband J Jacoby J Higgins R Crider D Thiopental vs. etomidate for rapid sequence intubation in aeromedicine Prehosp Disaster Med 2005 20 324 326 16295169 10.1017/s1049023x00002788

[B74] Swanson ER Fosnocht DE Jensen SC Comparison of etomidate and midazolam for prehospital rapid-sequence intubation Prehosp Emerg Care 2004 8 273 279 15295727 10.1016/j.prehos.2003.12.026

[B75] Tam RK Maloney J Gaboury I Verdon JM Trickett J Leduc SD Poirier P Review of endotracheal intubations by Ottawa advanced care paramedics in Canada Prehosp Emerg Care 2009 13 311 315 10.1080/10903120902935231 19499466

[B76] Swanson ER Fosnocht DE Effect of an airway education program on prehospital intubation Air Med J 2002 21 28 31 12087321 10.1067/mmj.2002.125936

[B77] Timmermann A Russo SG Eich C Roessler M Braun U Rosenblatt WH Quintel M The out-of-hospital esophageal and endobronchial intubations performed by emergency physicians.[see comment] Anesth Analg 2007 104 619 623 10.1213/01.ane.0000253523.80050.e9 17312220

[B78] Vadeboncoeur TF Davis DP Ochs M Poste JC Hoyt DB Vilke GM The ability of paramedics to predict aspiration in patients undergoing prehospital rapid sequence intubation J Emerg Med 2006 30 131 136 10.1016/j.jemermed.2005.04.019 16567245

[B79] Wang HE O'Connor RE Schnyder ME Barnes TA Megargel RE Patient status and time to intubation in the assessment of prehospital intubation performance Prehosp Emerg Care 2001 5 10 18 10.1080/10903120190940254 11194061

[B80] Wayne MA Friedland E Prehospital use of succinylcholine: a 20-year review Prehosp Emerg Care 1999 3 107 109 10.1080/10903129908958916 10225641

